# Association between type 2 diabetes and periodontitis: a population-based study in the North Peru

**DOI:** 10.12688/wellcomeopenres.23036.3

**Published:** 2024-11-26

**Authors:** Marcela Mayta-Mayorga, Victoria Guerra-Rodríguez, Antonio Bernabe-Ortiz

**Affiliations:** 1 Universidad Cientifica del Sur, Lima, Peru

**Keywords:** Periodontitis, periodontal disease, type 2 diabetes mellitus, prediabetic state.

## Abstract

**Background:**

Periodontitis, one of the most common forms of periodontal disease, has been linked to several cardiovascular factors including metabolic syndrome and inflammatory processes. This study aimed to determine the association between type 2 diabetes mellitus (T2DM) and periodontitis in a representative sample of individuals in the north of Peru.

**Materials and methods:**

Secondary data analysis using information of a population-based survey, enrolling subjects aged 35 to 69 years. The outcome was periodontitis, evaluated using a self-reported and validated 8-item questionnaire (≥5 points compatible with severe periodontitis), whereas the exposure was the presence of T2DM, evaluated using results of oral glucose tolerance test and categorized into two different forms: (a) normoglycemic, prediabetes, and T2DM, and (b) without T2DM, with T2DM and <5 years of diagnosis, and with T2DM and ≥5 years of diagnosis. Poisson regression models were utilized to report prevalence ratios (PR) and 95% confidence intervals (95% CI).

**Results:**

Data from 1606 individuals were analyzed, with a mean age of 48.2 (SD: 10.6) years, and 50.3% were women. Of these, 272 (16.9%) had prediabetes and 176 (11.0%) had T2DM (71.6% with <5 years of disease). Overall, 97.0% presented at least one symptom compatible with periodontitis, 882 (55.0%) had mild, 643 (40.0%) had moderate, and 5% had severe periodontitis. In multivariable model, those with T2DM had a higher prevalence of severe periodontitis (PR = 1.99; 95% CI: 1.12 - 3.54). Similarly, those with <5 years of disease had a higher prevalence of severe periodontitis (PR = 2.48; 95% CI: 1.38 - 4.46).

**Conclusions:**

Our research confirms the association between T2DM and severe periodontitis, especially among those with recent diagnosis (<5 years). Symptoms of periodontitis are quite common in our study population. Our results suggest a need to periodically assess oral health in patients with T2DM.

## Plain Language Summary

Individuals with type 2 diabetes mellitus, mainly those with shorter duration of disease (i.e., < 5 years), presented high prevalence of periodontitis (i.e., gum disease). Additionally, a huge proportion of study subjects had at least one symptom of gum disease, highlighting this condition is relatively common in our population. Furthermore, our findings underpin the need of periodically assess oral health among subjects with alteration of the glucose metabolism.

## Introduction

Periodontitis is a common infectious disease with a prevalence of up to 50% worldwide ^1^, with an estimated 10% having severe periodontitis ^2^, and a total of 1.1 billion prevalent cases ^3^. A systematic review including 30 studies conducted in India reported a prevalence of periodontitis of 51% in adults aged 18 years and older ^4^. On the other hand, the severity of such condition increases with age, with about 19% of American adults ≥65 years being edentulous ^5^. In Peru, the prevalence of severe periodontitis in people ≥15 years has been estimated to be 19%, whilst the prevalence of edentulism is around 15% ^6^.

Periodontitis can be caused by many factors that may be non-modifiable (male sex, older age, and heredity, including genetic diseases) ^5, 7^, and modifiable factors, such as tobacco use, poor oral hygiene, type 2 diabetes mellitus, and pregnancy ^8– 11^. Periodontitis, which is the most common manifestation of periodontal disease, is described as the sixth complication of diabetes mellitus ^12^, and it is much more frequent among people with than those without diabetes (68% vs. 36%) ^13^. The conduction of studies to assess the prevalence of periodontitis in the general population requires direct evaluation of the oral cavity by a specialist. Nevertheless, currently, certain authors have reported validated scales that can be used to conduct epidemiological studies, reducing the time and cost of evaluating this condition ^1, 14^.

Several studies show a bidirectional association between type 2 diabetes mellitus (T2DM) and periodontitis, due to the inflammatory mechanism produced by both pathologies ^15, 16^. In addition, better glycemic control (e.g., reduction of glycosylated hemoglobin levels) has been reported three months after nonsurgical treatment of periodontitis ^17^. T2DM can increase the risk of developing periodontitis by 34%, but at the same time, severe periodontitis increases the incidence of T2DM by 53% ^12, 18^. However, there is lack of evidence focused on prediabetes, and few studies have been conducted in the general population and the existing ones had limitations in focusing on risk or interest groups (pregnant women), only diabetic patients without a comparison group, or a small group of study subjects. Moreover, very few studies have been conducted in resource-constrained settings.

Few studies have evaluated the prevalence of periodontitis in the adult population using a validated scale ^14, 19^, especially in the general population and in constrained-resource settings, such as Peru. Moreover, a more limited number of studies have evaluated the association between prediabetes and periodontitis using the gold standard for screening for T2DM (i.e., oral glucose tolerance test). Early detection of periodontitis may be important to provide treatment and adequate control that will prevent complications on other organs and tissues of the body. Therefore, this study aimed to evaluate the association between glycemic status, including prediabetes and type 2 diabetes mellitus, and periodontitis, using information of a large-scale population-based study conducted in northern Peru.

## Methods

### Study design

This is a secondary analysis of data from a population-based, cross-sectional study conducted in Tumbes, northern Peru. The main objective of the original study was to assess the diagnostic accuracy of the Finnish Diabetes Risk Score (FINDRISC) for T2DM diagnosis and to compare its performance with other risk scores ^20^. Data for this study were collected between December 2016 and November 2017. Report of this manuscript has followed the STROBE checklist ( *See Extended data*).

### Study site and population

The study was conducted in the area surrounding Tumbes, a region with an area of 4,669 km ^2^ and a population of nearly 225,000 inhabitants, located in the north of the Peruvian coast ^21^. The main productive activities in the region are agriculture (especially rice and banana), trade and manufacturing.

The original study recruited participants aged between 35 and 69 years, able to understand the procedures and give informed consent, and who lived full-time in the study area (≥6 months). Pregnant women and bedridden patients were excluded. For the present analysis, the same inclusion criteria of the original study were used; however, only those questionnaires that contained all the information on the main variables were considered, that is, complete information about periodontitis and measurements of fasting plasma glucose and postprandial glucose, to define the glycemic status.

### Sampling

A single-stage, random sampling strategy, stratified by sex, was used, taking into account the results of the last available census in the study area (2014). No more than one participant per household was recruited to prevent a possible clustering of behavioral factors.

Power Analysis and Sample Size (PASS) software was used for sample size calculation. PASS is a computer program, produced by NCSS LLC, for estimating sample size or determining the power of a statistical test or confidence interval. Assuming a significance level of 5%, with 1334 participants obtained by adding the two categories of interest (normoglycemia and T2DM), there was a power greater than 99% to detect a difference in the prevalence of severe periodontal disease between people with T2DM of 9.7% and in people without T2DM of 4.8%.

### Definition of variables

***Outcome:*** Periodontitis was defined using the Eke questionnaire (validated in Spanish) and assessed by self-report of oral health using eight questions ^22^. The decision to use the Eke questionnaire was based on its suitability for large-scale epidemiological studies as well as cost-effectiveness compared to specialists. Despite that, under-reporting and misclassification of cases may be an issue as it is based on self-reporting. Questions of this tool comprised information on gum disease, gum health status, previous gum treatment, assessed the degree of tooth detachment from the gum, the state of the bone around the teeth, self-reported appearance of the teeth, and frequency of use of adjuvants in oral health (i.e., floss use and mouthwash). The total score ranges from 0 to 8 and those with a score ≥5 points were classified as having severe periodontitis ^22^.

***Exposure:*** Glycemic status, evaluated using the oral glucose tolerance test and classifying participants into normal, prediabetes, and those with type 2 diabetes mellitus (T2DM) according to the international guidelines ^23^: those participants with fasting plasma glucose <100 mg/dl and postprandial glucose <140 mg/dl were classified as normal; those with fasting plasma glucose between 100 mg/dl to 125 mg/dl and/or postprandial glucose between 140 mg/dl to 199 mg/dl were classified as prediabetic; and those who had fasting plasma glucose results ≥126 mg/dl and/or postprandial glucose ≥200 mg/dl and/or those with a previous diagnosis were classified as having T2DM. In addition to that, participants were classified into those who did not have and those who had a history of T2DM; however, the second group was split into two groups according to the duration of the disease: <5 years and ≥5 years. In this way, the final variable had three categories: without T2DM, with T2DM and <5 years of disease, and with T2DM and ≥5 years of disease.

***Covariates:*** Other variables were used for descriptive purposes and as potential confounders, including: sex (male vs. female); age (<40, 40–49, 50–59, ≥60 years); education level (primary, secondary, higher); socioeconomic status, assessed through household possessions and then categorized into tertiles (low, medium, high); currently employed (no vs. yes); daily tobacco use (at least one cigarette per day, no vs. yes); alcohol consumption, based on the number of times the participant reported consuming at least 6 bottles of beer (or equivalent) on a single occasion (never, ≤1 time per month, >1 time per month); fruit and vegetable intake (at least one fruit or vegetable per day); processed sugar consumption reported in the past week (never, ≤1 time per week, >1 time per week); physical activity levels, based on the International Physical Activity Questionnaire and categorized according to the number of metabolic equivalents per minute in the past week (moderate/high vs. low); and body mass index, defined according to traditional cut-off points (normal [BMI <25 kg/m2], overweight [25<BMI<30 kg/m2], and obese [BMI≥30 kg/m2]) ^24^.

### Procedures

In a pilot study enrolling 30 patients with and 30 without T2DM, the procedures, order and time in which the evaluations would be given were organized. For example, the questionnaire and the anthropometric measurements were planned to be conducted between the two blood measurements (fasting and postprandial).

During the fieldwork, the households of potential participants were visited to invite them to take part in the study. A written informed consent was applied to participants before starting data collection. The information was collected using tablets, through an application created with Open Data Kit (ODK) and the measurements were taken by trained personnel ( *See Extended data*).

After the fasting period (8 to 12 hours) was verified, a 7.5-ml venous blood sample was taken to assess fasting glucose. After that, the participant ingested a 75-g anhydrous glucose load in a volume of 300 ml as recommended by international guidelines ^23^. After two hours, a new blood sample was drawn to measure postprandial glucose levels. Between the first and second blood draws, questionnaires were administered, as well as anthropometric measurements (height using a stadiometer and weight was assessed using a bioelectrical impedance device [TBF-300A body composition analyzer/scale and thermographic paper, capacity: 400 lbs., TANITA Corporation, Tokyo, Japan]).

Blood analyses were performed by a certified Peruvian laboratory located in Lima. Initially, a grey-top tube (2 ml) containing sodium fluoride EDTA (3mg/6mg) was used. After drawing blood, the tube was moved upside down 8 to 10 times to ensure homogeneity. Samples were then transported withing the next four hours to a local laboratory, where the samples were initially centrifuged to separate serum into cryovials and then frozen (-20°C) to be sent to Lima for analysis. Glucose was measured in serum using a Cobas modular platform automated analyzer and reagents (number of reagents used 3350, including fasting and postprandial assessments), supplied by Roche Diagnostics (catalogue number: 04404483190). Quality control for glucose measurements had <1 for the coefficient of variation, a reference range provided by Bio-Rad, an independent testing company ( www.biorad.com).

### Statistical analysis

Statistical analysis was performed using STATA version 16 for Windows (StataCorp, College Station, Texas, USA), and p values < 0.05 were considered statistically significant. To describe the study population, means and standard deviation (SD) or median and interquartile range (IQR) were used for numerical variables as appropriate; whilst frequencies and proportions were used for categorical variables. Prevalence and the respective 95% confidence intervals (95% CI) of the variables of interest were reported. Comparisons between variables were performed using the Chi-square test for categorical variables. Finally, to verify the association of interest, Poisson regression models were used, with robust variance, and with this, the prevalence ratio (PR) and its respective 95% CI were reported. Poisson regression with robust variance provides correct estimates and is a better alternative for the analysis of cross-sectional studies with binary outcomes than logistic regression. Multivariable models were adjusted for variables defined a priori, based on the literature (sex, age, education level, socioeconomic status, currently working, daily tobacco use, alcohol use, fruit and vegetable intake, processed sugar consumption, physical activity levels, and body mass index).

## Results

### Characteristics of the study population

A total of 1612 subjects were enrolled, but only 1609 completed all the procedures of the study. Of them, two records were excluded for not having complete oral glucose tolerance tests and one for not having periodontitis results, leaving a total of 1606 individuals, with a mean age of 48.2 (SD 10.6) years and 50.3% were women.

### Glycemic status and associated factors

Of the 1606 individuals analyzed, 272 (16.9%; 95%CI: 15.1% - 18.9%) had values compatible with prediabetes, and 176 (11.0%; 95%CI: 9.5% - 12.6%) were classified as having T2DM. Of the total number of individuals with T2DM, 126 (71.6%) had less than 5 years of disease. In bivariate analysis, age, educational level, currently working, alcohol consumption, physical activity level, consumption of processed sugars, and BMI were associated with glycemic status using both definitions ( Table 1).

**Table 1.** Characteristics of the study population according to glycemic status and duration of T2DM.

|	Normal	Prediabetes	T2DM	p *	No T2DM	T2DM <5 y	T2DM ≥5 y	p *
	(n = 1158)	(n = 272)	(n = 176)		(n = 1430)	(n = 126)	(n = 50)	
***Sex***								
Male	611 (52.8%)	112 (41.2%)	74 (42.1%)	<0.001	723 (50.6%)	53 (42.1%)	21 (42.0%)	0.10
Female	547 (47.2%)	160 (58.8%)	102 (57.9%)		707 (49.4%)	73 (57.9%)	29 (58.0%)	
*Age*								
<40 years	372 (32.1%)	54 (19.9%)	14 (7.9%)	<0.001	426 (29.8%)	13 (10.3%)	1 (2.0%)	<0.001
40–49 years	353 (30.5%)	81 (29.8%)	45 (25.6%)		434 (30.4%)	38 (30.2%)	7 (14.0%)	
50–59 years	264 (22.8%)	76 (27.9%)	69 (39.2%)		340 (23.8%)	47 (37.3%)	22 (44.0%)	
>60 years	169 (14.6%)	61 (22.4%)	48 (27.3%)		230 (16.0%)	28 (22.2%)	20 (40.0%)	
***Education level***								
Primary	336 (29.0%)	102 (37.5%)	80 (45.4%)	<0.001	438 (30.6%)	54 (42.9%)	26 (52.0%)	0.001
Secondary	558 (48.2%)	116 (42.7%)	73 (41.5%)		674 (47.1%)	55 (43.6%)	18 (36.0%)	
Superior	264 (22.8%)	54 (19.8%)	23 (13.1%)		318 (22.3%)	17 (13.5%)	6 (12.0%)	
***Socioeconomic status***								
Low	370 (32.0%)	100 (36.8%)	68 (38.6%)	0.30	470 (32.9%)	48 (38.2%)	20 (40.0%)	0.60
Middle	408 (35.2%)	89 (32.7%)	53 (30.1%)		497 (34.7%)	39 (30.9%)	14 (28.0%)	
High	380 (32.8%)	83 (30.5%)	55 (31.3%)		463 (32.4%)	39 (30.9%)	16 (32.0%)	
***Currently working***								
No	330 (28.5%)	109 (40.1%)	78 (44.3%)	<0.001	439 (30.7%)	52 (41.3%)	26 (52.0%)	<0.001
Yes	828 (71.5%)	163 (59.9%)	98 (55.7%)		991 (63.3%)	74 (58.7%)	24 (48.0%)	
***Daily tobacco use***								
No	1086 (93.8%)	260 (95.6%)	168 (95.5%)	0.40	1346 (94.1%)	121 (96.0%)	47 (94.0%)	0.68
Yes	72 (6.2%)	12 (4.4%)	8 (4.5%)		84 (5.9%)	5 (4.0%)	3 (6.0%)	
***Alcohol consumption***								
Never	453 (39.1%)	128 (47.0%)	104 (59.1%)	<0.001	581 (40.6%)	70 (55.6%)	34 (68%)	<0.001
≤1 time/month	577 (49.8%)	131 (48.2%)	61 (34.7%)		708 (49.5%)	46 (36.5%)	15 (30.0%)	
>1 time/ month	128 (11.1)	13 (4.8%)	11 (6.2%)		141 (9.9%)	10 (7.9%)	1 (2.0%)	
***Fruit and vegetable intake***								
<1 per day	547 (47.2%)	133 (48.9%)	86 (48.9%)	0.84	680 (47.6%)	62 (49.2%)	24 (48.0%)	0.94
≥1 per day	611 (52.8%)	139 (51.1%)	90 (51.1%)		750 (52.4%)	64 (50.8%)	26 (52.0%)	
***Consumption of processed sugars***								
Never	561 (48.4%)	128 (47.1%)	119 (67.6%)	<0.001	689 (48.2%)	80 (63.5%)	39 (78.0%)	<0.001
≤1 time/week	376 (32.5%)	103 (37.9%)	33 (18.8%)		479 (33.5%)	24 (19.0%)	9 (18.0%)	
>1 time/week	221 (19.1%)	41 (15.0%)	24 (13.6%)		262 (18.3%)	22 (17.5%)	2 (4.0%)	
***Physical activity levels***								
Moderate/high	743 (64.2%)	165 (60.7%)	94 (53.4%)	0.019	908 (63.5%)	74 (58.7%)	20 (40.0%)	0.002
Low	415 (35.8%)	107 (39.3%)	82 (46.6%)		522 (36.5%)	52 (41.3%)	30 (60.0%)	
***Body mass index***								
Normal	341 (29.5%)	47 (17.3%)	37 (21.0%)	<0.001	388 (27.1%)	20 (15.9%)	17 (34.0%)	0.06
Overweight	514 (44.4%)	111 (40.8%)	81 (46.0%)		625 (43.7%)	62 (49.2%)	19 (38.0%)	
Obesity	303 (26.1%)	114 (41.9%)	58 (33.0%)		417 (29.2%)	44 (34.9%)	14 (28.0%)	

* Comparisons were done using Chi-squared test

### Periodontal disease and associated factors

In the study population, 97.0% presented at least one symptom compatible with periodontitis. The prevalence of severe periodontitis was (5.0%; 95% CI: 4.0% - 6.2%), whereas 882 (55.0%) of the cases reported mild periodontitis, and 643 (40.0%) were categorized as moderate periodontitis. Both daily tobacco use, and glycemic status were factors associated with the presence of severe periodontitis ( Table 2).

**Table 2.** Characteristics of the study population by severe periodontal disease.

Variables	Without severe PD	With severe PD	p *
	(n=1525)	(n = 81)	
***Sex***			
Male	758 (49.7%)	39 (48.2%)	0.79
Female	767 (50.3%)	42 (51.8%)	
***Age***			
<40 years	426 (27.9%)	14 (17.3%)	0.131
40–49 years	455 (29.8%)	24 (29.6%)	
50–59 years	385 (25.3%)	24 (29.6%)	
>60 years	259 (17.0%)	19 (23.5%)	
***Education level***			
Primary	485 (31.8%)	33 (40.7%)	0.20
Secondary	712 (46.7%)	35 (43.2%)	
Superior	328 (21.5%)	13 (16.1%)	
***Socioeconomic status***			
Low	511 (33.5%)	27 (33.3%)	0.99
Middle	522 (34.2%)	28 (34.6%)	
High	492 (32.3%)	26 (32.1%)	
***Currently working***			
No	490 (32.1%)	27 (33.3%)	0.82
Yes	1035 (67.9%)	54 (66.7%)	
***Daily tobacco use***			
No	1142 (94.6%)	72 (88.9%)	0.03
Yes	83 (5.4%)	9 (11.1%)	
***Alcohol consumption***			
Never	653 (42.8%)	32 (39.5%)	0.59
≤1 time/month	726 (47.6%)	43 (53.1%)	
>1 time/ month	146 (9.6%)	6 (7.4%)	
***Fruit and vegetable intake***			
<1 per day	727 (47.7%)	39 (48.2%)	0.93
≥1 per day	798 (52.3%)	42 (51.8%)	
***Consumption of processed sugars***			
Never	772 (50.6%)	36 (44.5%)	0.14
≤1 time/week	488 (32.0%)	24 (29.6%)	
>1 time/week	265 (17.4%)	21 (25.9%)	
***Physical activity levels***			
Moderate/high	952 (62.4%)	50 (61.7%)	0.90
Low	573 (37.6%)	31 (38.3%)	
***Body mass index***			
Normal	399 (26.2%)	26 (32.1%)	0.13
Overweight	667 (43.7%)	39 (48.1%)	
Obesity	459 (30.1%)	16 (19.8%)	
***Glycemic status***			
Normal	1103 (72.3%)	55 (67.9%)	0.008
Prediabetes	263 (17.3%)	9 (11.1%)	
T2DM	159 (10.4%)	17 (21.0%)	
***Duration of disease***			
No T2DM	1366 (89.6%)	64 (79.0%)	0.005
T2DM <5 years	112 (7.3%)	14 (17.3%)	
T2DM ≥5 years	47 (3.1%)	3 (3.7%)	

PD: Periodontal disease * Comparisons were done using Chi-squared test

### Association between type 2 diabetes mellitus and severe periodontal disease

In the multivariate model, adjusted for sex, age, education level, socioeconomic status, current employment, daily tobacco use, alcohol consumption, fruit and vegetable intake, processed sugar consumption, physical activity levels, and body mass index, T2DM was associated with severe periodontitis (PR = 1.99; 95% CI: 1.12 - 3.54). However, prediabetes was not associated with the outcome of interest ( Table 3).

**Table 3.** Association between T2DM and severe periodontal disease: Crude and adjusted models.

	Crude model	Adjusted model *
	PR (95% CI)	PR (95% CI)
**Glycemic status**		
Normal	1 (Reference)	1 (Reference)
Prediabetes	0.70 (0.35- 1.39)	0.71 (0.36 - 1.42)
T2DM	2.03 (1.21 - 3.42)	**1.99 (1.12 - 3.54)**
**Duration of disease**		
No T2DM	1 (Reference)	1 (Reference)
T2DM <5 years	2.48 (1.43 - 4.30)	**2.48 (1.38 - 4.46)**
T2DM ≥5 years	1.34 (0.44 - 4.12)	1.23 (0.39 - 3.91)

T2DM: Type 2 diabetes mellitus; PR: prevalence ratio; 95% CI: 95% confidence intervals * Model adjusted for sex, age, education level, socioeconomic status, currently working, daily tobacco use, alcohol consumption, fruit and vegetable intake, and body mass index.

When the definition of glycemic status considering disease duration was used ( Table 3), those with a shorter duration of T2DM (< 5 years since diagnosis) had a higher prevalence of severe periodontitis (PR = 2.48; 95% CI: 1.38 - 4.46) compared to those with normal glycemia. However, there was no association in those with a longer duration of disease (PR = 1.23; 95% CI: 0.39 - 3.91).

## Discussion

### Main findings

According to the results of the present study, there is a direct association between T2DM, but not prediabetes, and severe periodontitis. On the other hand, those individuals with a shorter duration of disease (<5 years) are more likely to have severe periodontitis than those with a longer duration of disease, which could be associated with severe periodontitis being a predictive condition for T2DM ^12^. Finally, almost all participants presented some degree of periodontitis, but only 5% had severe patterns of the condition, and more than 10% of study subjects had results compatible with T2DM.

### Comparison with previous studies

Several observational and experimental studies have shown that periodontitis can impact systemic health through various molecular mechanisms ^12, 13, 25^. An independent connection has been established between periodontitis and most chronic systemic diseases, including metabolic syndrome, which involves elevated glucose levels ^26^.

Literature demonstrates a bidirectional relationship between periodontitis, the most common presentation of periodontal disease, and T2DM ^12, 16, 27^. For example, in a systematic review, the results of longitudinal studies reported that T2DM could increase the risk of developing periodontitis by 34%, while severe periodontitis increased the incidence of T2DM by 53% ^12^. The same study reported that the impact of mild periodontitis on the incidence of T2DM was significant, although less robust; and that those with T2DM had deeper periodontal pockets and greater tooth loss compared to those without T2DM. Another meta-analysis supports such an association ^28^, indicating a positive bidirectional association between periodontitis and T2DM and, therefore, underscores the need for screening patients with periodontitis for T2DM and vice versa. Finally, another meta-analysis reported the effect of scaling and root planning on glycemic and inflammatory control in patients with T2DM with periodontitis ^17^. Data from nine clinical trials were analyzed, with low levels of heterogeneity, and it was shown that scaling and root planning was effective in reducing levels of glycosylated hemoglobin and C-reactive protein.

### Relevance of results

Our findings demonstrate the need for a complete evaluation of individuals with T2DM, especially in the case of oral health. The presence of periodontal disease symptoms is almost universal, and a large percentage of subjects in the study presents moderate and severe levels of periodontitis. These findings, together with the data in the literature, also show that periodontal treatment can help to better control glycemic and inflammatory conditions in patients with T2DM, with the subsequent clinical impact to avoid complications.

On the other hand, the inflammatory process of periodontitis, especially in severe cases, can increase the risk of developing T2DM. Our results may evidence that as cases with T2DM with less disease duration presented a greater prevalence of periodontitis. Therefore, the surveillance and treatment of periodontitis and periodontal disease may be relevant to reduce the risk of metabolic complications. Preventive guidelines in Peru should emphasize this fact by ensuring appropriate screening and management of periodontitis, especially in those with T2DM ^29^.

### Strengths and limitations

This is a population-based survey utilizing the oral glucose tolerance test to define T2DM, the gold standard for that diagnosis. In addition, a self-reported scale, validated in Spanish, was used to assess periodontitis. Despite these strengths, the study has some limitations that deserve mention. First, being a cross-sectional study, it can only assess association, but not causality. However, the existing literature is consistent with our findings. Second, although a validated scale was used, self-reported symptoms were used to define periodontitis. Nevertheless, our findings suggest the need to incorporate clinical or radiographic assessments in future studies. Third, there is a potential selection bias, since the study was conducted using a sample of participants aged 35 to 69 years in a semiurban area surrounding the city of Tumbes, an area with a high prevalence of T2DM and other risk factors, so our results may be limited to that population group. Moreover, there could also be recall bias because certain questions are about past and not recent topics (i.e., alcohol consumption). Fourth, despite selecting only one participant per household, some residual clustering effect may be present due to communal influences on lifestyle, dental hygiene practices, or dietary habits. Finally, being a secondary data analysis, certain variables of interest were not available, such as tooth brushing history and frequency, oral hygiene, and other related variables.

## Conclusions

Our research confirms the association between T2DM and periodontitis. Periodontitis symptoms are quite common in our study population. Our results suggest a need for periodic assessment of oral health in patients with T2DM.

## Ethics and consent

The present study adhered to the Declaration of Helsinki. The protocol and informed consent were approved by the Ethics Committee of the Universidad Peruana Cayetano Heredia, in Lima, Peru (SIDISI code 63585, date of approval: February 10, 2015) and the London School of Hygiene & Tropical Medicine, London, United Kingdom (code: 11783, date of approval: October 3, 2016). A written informed consent was read before enrolment to ensure participation. The present analysis was reviewed and approved by the Ethics Committee of the Universidad Científica del Sur (PRE-15-2022-00368).

## Data Availability

Figshare: T2DM SCREEN baseline for “Association between type 2 diabetes and periodontitis: a population-based study in the North Peru”. https://doi.org/10.6084/m9.figshare.26493139.v1 ^30^ This project contains the following underlying data: - T2DM SCREEN v11.csv (dataset) - Dictionary (110521).txt (key to variable abbreviations) Data are available under the terms of the Creative Commons Attribution 4.0 International license (CC-BY 4.0). Figshare: T2DM SCREEN – Questionnaires (baseline and follow-up) for “Association between type 2 diabetes and periodontitis: a population-based study in the North Peru”. https://doi.org/10.6084/m9.figshare.26970388.v1 ^31^. Data are available under the terms of the Creative Commons Attribution 4.0 International license (CC-BY 4.0).

## Software availability

Open Data Kit (ODK) for data collection, a free software at the moment of the survey, currently it is not. Available at: https://getodk.org/.
