# Supplementary material for: Association between type 2 diabetes and periodontitis: a population-based study in the North Peru
Source: Wellcome Open Res. 2024 Dec 24;9:562. Originally published 2024 Oct 2. [Version 4] doi: 10.12688/wellcomeopenres.23036.4 (PMC11586918; doi:10.12688/wellcomeopenres.23036.4)
Supplement: Peer review history

# Reviewer report: Reviewer response for version 4

**DOI:** 10.21956/wellcomeopenres.25972.r117708

**Published:** 2025-02-04

**Authors:** Rohaida Abdul Halim

**Affiliations:** 1 Universiti Teknologi MARA, Shah Alam, Selangor, Malaysia

**Study design and methodology:**

- The population-based cross-sectional design, is appropriate for assessing the association between glycaemic status and periodontitis.
- The random sampling strategy and use of the oral glucose tolerance test (OGTT) strengthen the reliability of the glycaemic status classification.
- The use of the Poisson regression model with robust variance is a sound statistical approach for cross-sectional data with binary outcomes.

** However:-**

- The reliance on a self-reported measure for periodontitis (Eke questionnaire), though validated, introduces the potential for misclassification and underreporting. It would be better if authors could provide additional justification for selecting the Eke questionnaire over clinical examination.
- The cross-sectional nature of the study prevents causal inference.
- The study focuses on a semi-urban Peruvian population, which may limit generalizability to other populations.

**Conclusion**

- The conclusion aligns with the results and is supported by the statistical findings.
- The study highlights the need for oral health screening in T2DM patients, which is a valuable clinical implication.

**Limitations:**

- The findings on disease duration and periodontitis prevalence should be interpreted cautiously, given the cross-sectional design.
- The conclusion could better acknowledge the limitations of self-reported data and potential recall bias.

This study contributes valuable insights into the association between T2DM and periodontitis in a Latin American population, reinforcing the importance of interdisciplinary approaches in managing metabolic and oral health conditions.

Is the work clearly and accurately presented and does it cite the current literature?

Yes

If applicable, is the statistical analysis and its interpretation appropriate?

Yes

Are all the source data underlying the results available to ensure full reproducibility?

Yes

Is the study design appropriate and is the work technically sound?

Partly

Are the conclusions drawn adequately supported by the results?

Yes

Are sufficient details of methods and analysis provided to allow replication by others?

Yes

Reviewer Expertise:

T1DM and periodontal disease, paediatric dentistry

I confirm that I have read this submission and believe that I have an appropriate level of expertise to confirm that it is of an acceptable scientific standard.

# Reviewer report: Reviewer response for version 4

**DOI:** 10.21956/wellcomeopenres.25972.r117039

**Published:** 2025-01-17

**Authors:** Andjela Milojevic Samanovic

**Affiliations:** 1 Department of Dentistry, Faculty of Medical Sciences, University of Kragujevac, Kragujevac, Serbia

This cross-sectional observational study investigated the possible association between type 2 diabetes and periodontitis in a northern Peruvian population. According to the results of the present research, it is confirmed bidirectional relationship between the previously mentioned diseases.

 The study determined a significant association between periodontitis and glycaemic status, where the individuals with a shorter duration of disease (<5 years) had more severe periodontitis than those with a longer duration of disease.

 There are no aspects of the study that I have not been able to assess.

 Good investigation with good presentation of details with not so focused objective(s). However, the methodology is appropriate for this objective. At this stage, after this research, a good note on the limitations of this research with future directions at the end of the discussion may help the prospective readers and researchers.

 In addition, additional corrections are necessary:

1. Correct the mismatch of results in Tables 1 and 2, with the written text in the results section.
2. The author should explain, in the discussion section, the molecular mechanism of the relationship between type 2 diabetes and periodontitis.

Is the work clearly and accurately presented and does it cite the current literature?

Yes

If applicable, is the statistical analysis and its interpretation appropriate?

Yes

Are all the source data underlying the results available to ensure full reproducibility?

Yes

Is the study design appropriate and is the work technically sound?

Yes

Are the conclusions drawn adequately supported by the results?

Yes

Are sufficient details of methods and analysis provided to allow replication by others?

Yes

Reviewer Expertise:

relationship between periodontitis and diabetes

I confirm that I have read this submission and believe that I have an appropriate level of expertise to confirm that it is of an acceptable scientific standard.

# Reviewer report: Reviewer response for version 4

**DOI:** 10.21956/wellcomeopenres.25972.r116693

**Published:** 2024-12-30

**Authors:** He Lu

**Affiliations:** 1 Peking University School and Hospital of Stomatology, Beijing, China

1. There are some writing errors, such as "root planing" (not "planning"), BMI (kg/m²) (where "2" is superscript), and "overweight BMI ≥ 25" (not ">25").
2. In the results, the author states that "prediabetes was not associated with the outcome of interest" and "those individuals with a shorter duration of disease are more likely to have severe periodontitis than those with a long duration of disease." I agree with this conclusion based on the study and current population. However, it may not be the only accurate result for the relationship between prediabetes and periodontitis, or between periodontitis and the duration of T2DM, due to variations in population characteristics or periodontitis parameter definitions. However, the author has not elaborated on why they obtained such results in the discussion or limitations section.

Is the work clearly and accurately presented and does it cite the current literature?

Yes

If applicable, is the statistical analysis and its interpretation appropriate?

I cannot comment. A qualified statistician is required.

Are all the source data underlying the results available to ensure full reproducibility?

Yes

Is the study design appropriate and is the work technically sound?

Yes

Are the conclusions drawn adequately supported by the results?

Yes

Are sufficient details of methods and analysis provided to allow replication by others?

Yes

Reviewer Expertise:

NA

I confirm that I have read this submission and believe that I have an appropriate level of expertise to confirm that it is of an acceptable scientific standard, however I have significant reservations, as outlined above.

# Reviewer report: Reviewer response for version 4

**DOI:** 10.21956/wellcomeopenres.25972.r116329

**Published:** 2024-12-30

**Authors:** Rosamma Joseph Vadakkekuttical

**Affiliations:** 1 Government Dental College (Affiliated to Kerala University of Health Sciences), Calicut, Kerala, India

The overall status of the manuscript is satisfactory.

Is the work clearly and accurately presented and does it cite the current literature?

Yes

If applicable, is the statistical analysis and its interpretation appropriate?

Partly

Are all the source data underlying the results available to ensure full reproducibility?

Yes

Is the study design appropriate and is the work technically sound?

Yes

Are the conclusions drawn adequately supported by the results?

Yes

Are sufficient details of methods and analysis provided to allow replication by others?

Partly

Reviewer Expertise:

Periodontics, Diabetes  and periodontitis relationship ,Platelet Rich Fibrin(PRF) and periodontal surgery

I confirm that I have read this submission and believe that I have an appropriate level of expertise to confirm that it is of an acceptable scientific standard.

# Reviewer report: Reviewer response for version 3

**DOI:** 10.21956/wellcomeopenres.25882.r113461

**Published:** 2024-12-13

**Authors:** Rosamma Joseph Vadakkekuttical

**Affiliations:** 1 Government Dental College (Affiliated to Kerala University of Health Sciences), Calicut, Kerala, India

**Summary:**

 Periodontitis is a common infectious disease associated with both nonmodifiable and modifiable factors such as type 2 diabetes mellitus. There is a bidirectional relationship between type 2 diabetes mellitus (T2DM) and periodontitis; however, studies on the relationship between prediabetes and periodontal disease are scarce. This population based cross-sectional study evaluated the association betweenT2DM, prediabetes, and periodontal disease in Peruvian population. Periodontitis was assessed by a self – report questionnaire and glycemic status was assessed by glucose tolerance test. This study showed an association between type 2 diabetes mellitus (T2DM) and severe periodontitis, particularly with a duration of diabetes of less than five years. No association was found between prediabetes and periodontitis.

1. In this study, data from 1606 participants were analyzed. Of these, 97% presented with at least one symptom compatible with periodontitis, while that 3% (48) participants had no periodontal disease. In table 2, the category  ‘without severe periodontitis’ (n=1525)included all  participants  except those with severe periodontitis. It   should be clarified that this category encompasses moderate and mild/no periodontal disease and this should be described in the result section to avoid any confusion.  
  OR ‘without severe periodontitis’ n=1525- 48=1477
2. In table 2 the percentage of severe periodontitis is higher (67.9%) in normal group compared to T2DM group (21%), with the latter being reflective of the normal cross-section of the population.
3. It is not clear why the ‘No T2DM’ category is included in table 2, under the duration of diabetes?
4. In this study, drug history was not considered. Insulin and oral antidiabetic drug could have influenced the severity of periodontal disease

Is the work clearly and accurately presented and does it cite the current literature?

Yes

If applicable, is the statistical analysis and its interpretation appropriate?

Partly

Are all the source data underlying the results available to ensure full reproducibility?

Yes

Is the study design appropriate and is the work technically sound?

Yes

Are the conclusions drawn adequately supported by the results?

Yes

Are sufficient details of methods and analysis provided to allow replication by others?

Partly

Reviewer Expertise:

Periodontics, Diabetes  and periodontitis relationship

I confirm that I have read this submission and believe that I have an appropriate level of expertise to confirm that it is of an acceptable scientific standard, however I have significant reservations, as outlined above.

# Response

**Published:** 2024-12-16

**Authors:** Antonio Bernabe-Ortiz

**Affiliations:** Universidad Cientifica del Sur, Peru

In this study, data from 1606 participants were analyzed. Of these, 97% presented with at least one symptom compatible with periodontitis, while 3% (48) participants had no periodontal disease. In table 2, the category “without severe periodontitis” (n=1525) included all participants except those with severe periodontitis. It   should be clarified that this category encompasses moderate and mild/no periodontal disease and this should be described in the result section to avoid any confusion or ‘without severe periodontitis’ n=1525- 48=1477

**Response:** We have modified the manuscript to clarify this point. In the methods section, now it reads: “ *The total score ranges from 0 to 8 and those with a score ≥5 points were classified as having severe periodontitis. Thus, comparison was conducted between those with severe periodontitis and those without severe periodontitis (i.e., moderate, mild and no periodontal disease)*”. Other modifications have been made accordingly.  

 In table 2 the percentage of severe periodontitis is higher (67.9%) in normal group compared to T2DM group (21%), with the latter being reflective of the normal cross-sectional of the population.

**Response:** The table 2 shows that among those with severe periodontitis, 67.9% had normal glycemia and 21.0% had T2DM. We agree with the reviewer that this is a result of the cross-sectional nature of the study. When comparing the prevalence of severe periodontitis by glycemia status, results showed that among normal glycemic subjects, 4.8% had severe periodontitis, whereas these estimates were 3.3% in those with prediabetes, and 9.7% among those with T2DM. We have added that in the results section: “ *In crude model, the prevalence of severe periodontitis was higher among those with T2DM (9.7%) compared to those with prediabetes (3.3%) and those with normal glycemia (4.8%)*”.  

 It is not clear why the ‘No T2DM’ category is included in table 2, under the duration of diabetes?

**Response:** The general idea was to compare those with prediabetes and T2DM to those with normal glycemia. The same idea was kept in the case of years since T2DM diagnosis. This decision was based on the sample size, and the lack of power to detect differences between those with T2DM with <5 and ≥5 years of disease. For transparency, we have shown that information as part of the results: “ *Finally, in multivariable model, those with T2DM ≥5 years since diagnosis had lower prevalence of severe periodontitis (PR = 0.53; 95% CI: 0.12 – 2.33) compared to those with T2DM <5 years; nevertheless, the result was not significant*”.  

 In this study, drug history was not considered. Insulin and oral antidiabetic drug could have influenced the severity of periodontal disease.

**Response: **We have added this information to the limitation section: “ *…and other variables related to T2DM management (insulin and oral antidiabetic medication) which can influence the severity of periodontal disease*. *Moreover, drug history was not available due to the secondary data analysis nature*”.

# Reviewer report: Reviewer response for version 3

**DOI:** 10.21956/wellcomeopenres.25882.r113353

**Published:** 2024-12-06

**Authors:** Xin Huang

**Affiliations:** 1 Sun Yat-sen University, Guangdong, China

I do not have any comment this time.

Is the work clearly and accurately presented and does it cite the current literature?

Yes

If applicable, is the statistical analysis and its interpretation appropriate?

I cannot comment. A qualified statistician is required.

Are all the source data underlying the results available to ensure full reproducibility?

Yes

Is the study design appropriate and is the work technically sound?

Partly

Are the conclusions drawn adequately supported by the results?

Yes

Are sufficient details of methods and analysis provided to allow replication by others?

Yes

Reviewer Expertise:

relationship between periodontitis and diabetes, immune response

I confirm that I have read this submission and believe that I have an appropriate level of expertise to confirm that it is of an acceptable scientific standard.

# Reviewer report: Reviewer response for version 2

**DOI:** 10.21956/wellcomeopenres.25799.r111204

**Published:** 2024-11-20

**Authors:** Xin Huang

**Affiliations:** 1 Sun Yat-sen University, Guangdong, China

The revised version is much better. Approved to be Indexed.

Is the work clearly and accurately presented and does it cite the current literature?

Yes

If applicable, is the statistical analysis and its interpretation appropriate?

I cannot comment. A qualified statistician is required.

Are all the source data underlying the results available to ensure full reproducibility?

Yes

Is the study design appropriate and is the work technically sound?

Partly

Are the conclusions drawn adequately supported by the results?

Yes

Are sufficient details of methods and analysis provided to allow replication by others?

Yes

Reviewer Expertise:

relationship between periodontitis and diabetes, immune response

I confirm that I have read this submission and believe that I have an appropriate level of expertise to confirm that it is of an acceptable scientific standard.

# Reviewer report: Reviewer response for version 1

**DOI:** 10.21956/wellcomeopenres.25369.r104000

**Published:** 2024-11-11

**Authors:** Isabel Gallardo

**Affiliations:** 1 University of Sevilla, Sevilla, Spain

It is a cross-sectional observational study on the association between glycaemic status and periodontitis in a rural population in northern Peru. Previous studies have shown a bidirectional relationship between T2DM and periodontitis, however, they have not focused on prediabetic individuals or the duration of T2DM. The study found a significant association between glycaemic status and periodontitis, with diabetic participants showing a higher prevalence and severity of periodontitis, especially those with shorter disease duration.

 The study design is adequate: sample size (1606 participants), sampling randomization, glycaemic assessment (oral glucose tolerance test), however recruiting only one participant per household and evaluating periodontitis through a validated self-report may introduce some biases into the study.There seems to be an error in the percentages of patients with T2DM < 5 years' duration, they do not coincide in summary and results. I would consider specifying that PD patients in Table 2 refer to those with severe periodontitis, as it leads to confusion.

Is the work clearly and accurately presented and does it cite the current literature?

Yes

If applicable, is the statistical analysis and its interpretation appropriate?

I cannot comment. A qualified statistician is required.

Are all the source data underlying the results available to ensure full reproducibility?

Yes

Is the study design appropriate and is the work technically sound?

Partly

Are the conclusions drawn adequately supported by the results?

Yes

Are sufficient details of methods and analysis provided to allow replication by others?

Yes

Reviewer Expertise:

periodontal disease

I confirm that I have read this submission and believe that I have an appropriate level of expertise to confirm that it is of an acceptable scientific standard.

# Response

**Published:** 2024-11-20

**Authors:** Antonio Bernabe-Ortiz

**Affiliations:** Universidad Cientifica del Sur, Peru

The study design is adequate: sample size (1606 participants), sampling randomization, glycaemic assessment (oral glucose tolerance test), however recruiting only one participant per household and evaluating periodontitis through a validated self-report may introduce some biases into the study.

***Response:*** We have added information regarding this point in the limitations section. Now it reads: “ *Second, although a validated scale was used, self-reported symptoms were used to define periodontitis. Our findings suggest the need to incorporate clinical or radiographic assessments in future studies”*. We have also added a limitation related to the selection of one participant per household: “ *Fourth, despite selecting only one participant per household, some residual clustering effect may be present due to communal influences on lifestyle, dental hygiene practices, or dietary habits*”.  

 There seems to be an error in the percentages of patients with T2DM < 5 years' duration, they do not coincide in summary and results.

***Response:*** *We have verified our results and corrected them in the abstract as requested.*

 I would consider specifying that PD patients in Table 2 refer to those with severe periodontitis, as it leads to confusion.

***Response:*** *We have added such information in different parts of the text and not only in Table 2. This includes Table 3 and text of the Results and Discussion section.*

# Reviewer report: Reviewer response for version 1

**DOI:** 10.21956/wellcomeopenres.25369.r102774

**Published:** 2024-10-27

**Authors:** Xin Huang

**Affiliations:** 1 Sun Yat-sen University, Guangdong, China

**Summary**

 This study examines the association between glycemic status, including prediabetes and type 2 diabetes mellitus (T2DM), and periodontitis in a northern Peruvian population. Periodontitis, a common inflammatory condition linked to diabetes, is suggested to have a bidirectional relationship with T2DM, where each condition can exacerbate the other. Previous studies have often lacked focus on prediabetic populations or did not use validated periodontitis screening tools in large, general populations. This research used data from a cross-sectional study in Tumbes, Peru, including 1606 participants aged 35–69, to assess periodontitis via a validated self-report questionnaire and glycemic status with oral glucose tolerance testing. The study found a significant association between glycemic status and periodontitis, with prediabetic and diabetic participants exhibiting higher periodontitis prevalence.

**Responses to Questions & Suggested Improvements**

**Introduction**

1. **Thoroughness and Contextualization**:The section lacks details on specific knowledge gaps in the Peruvian context, especially in resource-limited settings, which would help contextualize the study's location and population better.
**Recommendation**: Include explicit statements on the scarcity of research focusing on prediabetes and on large-scale studies using validated tools in the general population. This will underline the study's unique contribution and justify the need for its population-specific focus.

**Methods**

1. **Study Design and Cohort Selection**:
**Areas for Improvement**: The authors could elaborate on why the validated Eke questionnaire was chosen over clinical examination, despite its limitations in capturing objective clinical severity.
**Recommendation**: Provide rationale for using the Eke questionnaire, mentioning its strengths (e.g., cost-effectiveness and suitability for large-scale epidemiological studies) and its limitations. This transparency enhances the study's validity and may prevent reader bias regarding potential underreporting or misclassification of periodontitis cases. 
2. **Sampling Strategy and Power Analysis**:
**Area for Improvement**: The sampling method's limitation of one participant per household to avoid clustering may not fully address family or communal lifestyle impacts on periodontitis and diabetes.
**Recommendation**: Acknowledge any residual clustering effects that could remain due to communal influences on lifestyle, dental hygiene practices, or dietary habits. Considering these factors will bolster the validity of the findings by demonstrating awareness of the potential limitations in study design.
3. **Definition of Variables**
**Operationalization of Variables:**
**Positive Aspect: **The clear definitions for periodontitis and glycemic categories are helpful, especially with detailed criteria.
**Area for Improvement**: The study's choice to define periodontitis based solely on a questionnaire without clinical confirmation could limit the diagnostic accuracy for mild cases.
**Recommendation**: Address the limitations of self-reported periodontitis diagnosis, suggesting that future studies incorporate clinical or radiographic assessments. This would strengthen the evidence base for the questionnaire's use in populations with potential low health literacy or access to dental care.
4. **Blood Sampling Protocol and Quality Control**:
**Positive Aspect**: Blood sample protocols, including fasting glucose testing, quality controls, and procedural details, are well explained.
**Area for Improvement**: Some readers may benefit from additional information on the logistics and timing of blood sample processing, particularly given the study site's resource constraints.
**Recommendation**: Consider adding specifics on transport, handling, and storage procedures for blood samples. This will provide insight into data reliability and address potential concerns regarding sample integrity, especially for the fasting and postprandial measurements in a field setting.
5. **Appropriateness of Statistical Methods**:
**Positive Aspect**: Statistical analyses, including descriptive statistics and regression models, are appropriate for the study objectives.
**Area for Improvement**: It is not entirely clear why Poisson regression was chosen over logistic regression, which is more conventional for prevalence studies.
**Recommendation**: Include a brief justification for using Poisson regression, noting its suitability for rare outcomes or count-based data. This addition will clarify any perceived methodological inconsistencies and highlight the robustness of the analytical approach.

Is the work clearly and accurately presented and does it cite the current literature?

Yes

If applicable, is the statistical analysis and its interpretation appropriate?

I cannot comment. A qualified statistician is required.

Are all the source data underlying the results available to ensure full reproducibility?

Yes

Is the study design appropriate and is the work technically sound?

Partly

Are the conclusions drawn adequately supported by the results?

Yes

Are sufficient details of methods and analysis provided to allow replication by others?

Yes

Reviewer Expertise:

relationship between periodontitis and diabetes, immune response

I confirm that I have read this submission and believe that I have an appropriate level of expertise to confirm that it is of an acceptable scientific standard, however I have significant reservations, as outlined above.

# Response

**Published:** 2024-11-07

**Authors:** Antonio Bernabe-Ortiz

**Affiliations:** Universidad Cientifica del Sur, Peru

**Introduction** **Thoroughness and Contextualization**: The section lacks details on specific knowledge gaps in the Peruvian context, especially in resource-limited settings, which would help contextualize the study's location and population better.

**Recommendation**: Include explicit statements on the scarcity of research focusing on prediabetes and on large-scale studies using validated tools in the general population. This will underline the study's unique contribution and justify the need for its population-specific focus.

***Response:*** We have rewritten the last paragraph of the Introduction to explicitly include the contribution of the study. Now it reads: “ *Few studies have evaluated the prevalence of periodontitis in the adult population using a validated scale, especially in the general population and in constrained-resource settings, such as Peru. Moreover, a more limited number of studies have evaluated the association between prediabetes and periodontitis using the gold standard for screening for T2DM (i.e., oral glucose tolerance test). Early detection of periodontitis may be important to provide treatment and adequate control that will prevent complications on other organs and tissues of the body. Therefore, this study aimed to evaluate the association between glycemic status, including prediabetes and type 2 diabetes mellitus, and periodontitis, using information of a large-scale population-based study conducted in northern Peru”.*  

**Methods** **Study Design and Cohort Selection**: **Areas for Improvement**: The authors could elaborate on why the validated Eke questionnaire was chosen over clinical examination, despite its limitations in capturing objective clinical severity.           

**Recommendation**: Provide rationale for using the Eke questionnaire, mentioning its strengths (e.g., cost-effectiveness and suitability for large-scale epidemiological studies) and its limitations. This transparency enhances the study's validity and may prevent reader bias regarding potential underreporting or misclassification of periodontitis cases. 

***Response:*** We have added the recommendation of the reviewer as follows: “ *The decision to use the Eke questionnaire was based on its suitability for large-scale epidemiological studies as well as cost-effectiveness compared to specialists. Despite that, under-reporting and misclassification of cases may be an issue as it is based on self-reporting*”.  

**Sampling Strategy and Power Analysis**: **Area for Improvement**: The sampling method's limitation of one participant per household to avoid clustering may not fully address family or communal lifestyle impacts on periodontitis and diabetes.

**Recommendation**: Acknowledge any residual clustering effects that could remain due to communal influences on lifestyle, dental hygiene practices, or dietary habits. Considering these factors will bolster the validity of the findings by demonstrating awareness of the potential limitations in study design.

***Response:*** We have added this to the limitation section as follows: “ *Fourth, despite selecting only one participant per household, some residual clustering effect may be present due to communal influences on lifestyle, dental hygiene practices, or dietary habits*”.  

**Definition of Variables** **Operationalization of Variables:** **Positive Aspect: **The clear definitions for periodontitis and glycemic categories are helpful, especially with detailed criteria.

**Area for Improvement**: The study's choice to define periodontitis based solely on a questionnaire without clinical confirmation could limit the diagnostic accuracy for mild cases. **Recommendation**: Address the limitations of self-reported periodontitis diagnosis, suggesting that future studies incorporate clinical or radiographic assessments. This would strengthen the evidence base for the questionnaire's use in populations with potential low health literacy or access to dental care.

***Response:*** We have expanded the limitation section accordingly. Now it reads: “ *Second, although a validated scale was used, self-reported symptoms were used to define periodontitis. Nevertheless, our findings suggest the need to incorporate clinical or radiographic assessments in future studies*”.   

**Blood Sampling Protocol and Quality Control**: **Positive Aspect**: Blood sample protocols, including fasting glucose testing, quality controls, and procedural details, are well explained. **Area for Improvement**: Some readers may benefit from additional information on the logistics and timing of blood sample processing, particularly given the study site's resource constraints. **Recommendation**: Consider adding specifics on transport, handling, and storage procedures for blood samples. This will provide insight into data reliability and address potential concerns regarding sample integrity, especially for the fasting and postprandial measurements in a field setting.

***Response:*** We have added the information requested: “ *Blood analyses were performed by a certified Peruvian laboratory located in Lima. Initially, a grey-top tube (2 ml) containing sodium fluoride EDTA (3mg/6mg) was used. After drawing blood, the tube was moved upside down 8 to 10 times to ensure homogeneity. Samples were then transported withing the next four hours to a local laboratory, where the samples were initially centrifuged to separate serum into cryovials and then frozen (-20°C) to be sent to Lima for analysis*”.

**Appropriateness of Statistical Methods**: **Positive Aspect**: Statistical analyses, including descriptive statistics and regression models, are appropriate for the study objectives. 

**Area for Improvement**: It is not entirely clear why Poisson regression was chosen over logistic regression, which is more conventional for prevalence studies.

**Recommendation**: Include a brief justification for using Poisson regression, noting its suitability for rare outcomes or count-based data. This addition will clarify any perceived methodological inconsistencies and highlight the robustness of the analytical approach. ***Response:*** According to Barros and Hirakata, Poisson regression with robust variance (and log-binomial regression) provides correct estimates and is a better alternative for the analysis of cross-sectional studies with binary outcomes than logistic regression, since the prevalence ratio is more interpretable and easier to communicate to non-specialists than the odds ratio (OR). Moreover, OR can overestimate the prevalence ratio, the measure of choice in these studies. We have added part of this as suggested: “ *Poisson regression with robust variance provides correct estimates and is a better alternative for the analysis of cross-sectional studies with binary outcomes than logistic regression*”.

***Reference:*** Barros AJD, Hirakata VN. BMC Med Res Methodol 2003; 3:21. doi: https://doi.org/10.1186/1471-2288-3-21
